# Osteopontin and Transplantation: Where Are We Now?

**DOI:** 10.1007/s00005-021-00617-6

**Published:** 2021-05-21

**Authors:** Beata Kaleta

**Affiliations:** Department of Clinical Immunology, Medical University of Warsaw, Nowogrodzka 59 St., 02-006 Warsaw, Poland

**Keywords:** Immunomodulation, Osteopontin, Rejection, Transplantation

## Abstract

Organ transplantation represents the optimal therapeutic tool for patients with end-stage organ failure. Hematopoietic stem cell transplantation (HSCT) is likewise an effective therapy for a wide range of malignant and non-malignant diseases. Better understanding of transplantation immunology and the use of multi-modal immunosuppression protocols, can decrease the risk of graft failure and graft-versus-host disease (GVHD) after HSCT. Nevertheless, a major challenge of modern transplantology still seems to be finding non-invasive biomarkers for recipients selection, monitoring of allograft function, and diagnosis of rejection. Since proinflammatory cytokine osteopontin (OPN) is closely involved in regulating both adaptive and innate immune responses, as well as the pathogenesis of inflammatory and autoimmune diseases, it is likely to play an important role in organ and HSC transplantation. This review is to summarize recent advances in our knowledge about OPN function in the kidney, heart, liver, lung, and HSC transplantation. Most studies found that elevated OPN is associated with poorer graft function in kidney, heart, liver and lung recipients. Moreover, some reports suggested that this protein can play role in GVHD pathogenesis. However, due to relatively small number of similar studies, as well as some inconclusive results, future investigation in this field is needed to verify if OPN can serve as a biomarker of organ and HSC transplantation. The knowledge about such markers will promote our understanding of the mechanisms underlying graft dysfunction and posttransplant mortality. In addition, such knowledge may be helpful in the development of new treatment strategies and identification of recipients with increased risk of allograft failure.

## Introduction

Organ transplantation is the gold standard therapy for advanced and irreversible kidney, liver, lung, as well as heart failure. Allogeneic hematopoietic stem cell transplantation (HSCT) is likewise the most beneficial therapeutic option for numerous malignant and non-malignant diseases (Mahmud et al. 2010). Recent advantages in immunological studies of donor–recipient pairs, and in immunosuppressive therapy reduced the risk of graft failure or graft-versus-host disease (GVHD) after HSCT. However, one of the major challenges of modern transplantology still seems to be finding non-invasive, prognostic and predictive biomarkers for recipients selection, monitoring of allograft function, and diagnosis of rejection (Naesens and Anglicheau 2018). Several studies have associated increased cytokine production with higher risk of early and late graft rejection and suggested that these proteins may serve as markers for kidney, liver, lung, heart and HSC transplantation (Chen et al. 2019; Friedman et al. 2012; Hallsten and Vigneswaran 2019; Kawakita and Everly 2016; Roedder et al. 2011; Visentainer et al. 2003). It is well documented that cytokines modulate the T helper (Th)1 and Th2 balance. In general, Th1 lymphocytes secrete interleukin (IL)-2, tumor necrosis factor (TNF)-α and interferon (IFN)-γ, and are responsible for macrophage activation. In general, Th2 lymphocytes produce IL-4, IL-5, IL-6, IL-10, and IL-13 and participate in antibody synthesis (Dallman 1995; Liang et al. 2003). Numerous studies have demonstrated that deregulation in the Th1/Th2 response may be associated with different mechanisms of rejection and graft function (Liang et al. 2003; Mota et al. 2013).

Osteopontin (OPN), a proinflammatory cytokine expressed in numerous cells and tissues, including activated T cells, macrophages, dendritic cells (DCs) and natural killer (NK) cells, has been studied in the context of various chronic diseases (Ashkar et al. 2000; Castello et al. 2017; Clemente et al. 2016; Icer and Gezmen-Karadag 2018; Lund et al. 2009). It has been found that this glycoprotein plays a significant role in immunomodulation and is involved in the pathogenesis of inflammatory and autoimmune diseases, such as multiple sclerosis, rheumatoid arthritis, systemic lupus erythematosus, cancer, atherosclerosis, chronic liver and kidney diseases (Agah et al. 2018; Ashkar et al. 2000; Ding et al. 2016; Kaleta 2014, 2019a; Lamort et al. 2019; Song et al. 2021; Zhang et al. 2015), and many others. In addition, some recent reports suggested that OPN may be associated with allograft failure as well as GVHD pathogenesis after HSCT. In this review I will discuss the role of OPN in both, organ and allogeneic HSC transplantation.

## Structure and Function of OPN

OPN is a phosphoglycoprotein also known as secreted phosphoprotein 1 (SPP-1) or early T lymphocyte activation-1. OPN is coded by the *SPP1* gene, located on chromosome 4q13 in humans (Icer and Gezmen-Karadag 2018). Its molecular weight varies between 44 and 75 kDa, which as documented, is associated with numerous post-translational modifications (phosphorylation, O-linked glycosylation, tyrosine sulfation, transglutamination, sialylation), which regulate and alter OPN function (Christensen et al. 2008). Moreover, it has been shown that OPN pre-mRNA splicing leads to the generation of five isoforms: OPN-a (full-length), OPN-b (lacks exon 5), OPN-c (lacks exon 4), OPN-4 (lacks exon 4 and 5), and OPN-5 (with an extra exon between exon 3 and 4) with different functional activities in physiological and pathological processes (Gimba et al. 2019; Young et al. 1990).

**Fig. 1:**
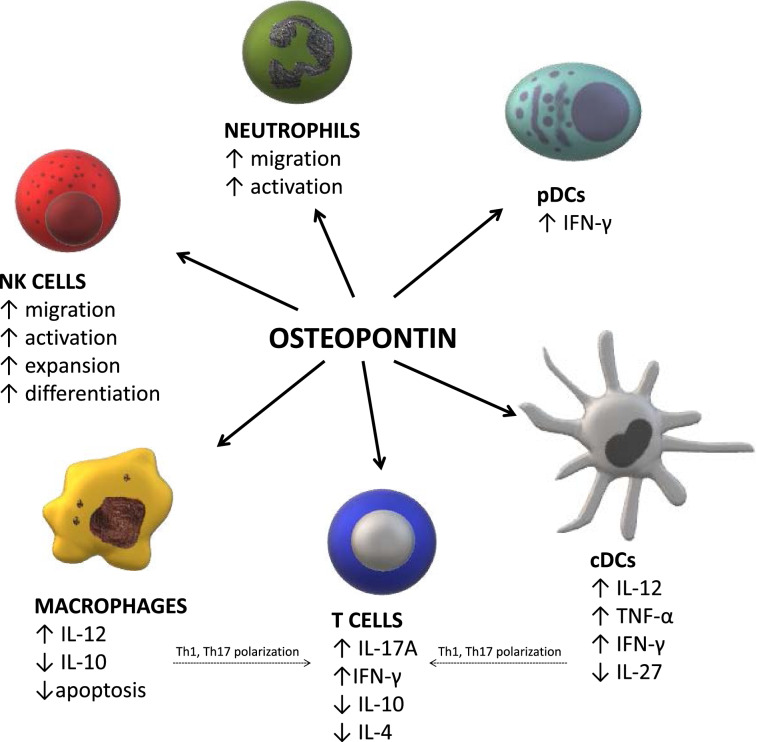
Osteopontin (OPN) effects on innate and adaptive immunity. In macrophages OPN upregulates interleukin (IL)-12 and downregulates IL-10 synthesis which results in T helper (Th)1 and Th17 polarization. In Th cells OPN enhances IL-17A and interferon (IFN)-γ expression and inhibits IL-4 and IL-10. In conventional dendritic cells (cDCs) OPN upregulates IL-12, tumor necrosis factor (TNF)-α and IFN-γ, and inhibits IL-27 production, which likewise contributes to enhancement of the Th1 and Th17 response. It also acts on plasmacytoid DCs (pDCs), inducing IFN-α synthesis. OPN acts on neutrophils, increasing their migration and activation. OPN increases natural killer (NK) cells activation, migration, expansion, as well as differentiation

OPN, one of the two non-collagenous proteins in bone was originally considered to act as a regulator of mineral metabolism (Kruger et al. 2014); however, later, it has been also identified as a mediator of innate-adaptive immune crosstalk (Fig. 1). OPN’s pleiotropic effects are partly due to its capacity to interact with multiple ligands including integrins and CD44 receptor (Clemente et al. 2016). In addition, recent studies have shown that this protein is a ligand for ICOSL (CD275) (Raineri et al. 2020). By interacting with α4 and α9 integrins, as well as with the CD44 receptor, OPN inhibits macrophage apoptosis, and stimulates their differentiation, migration, and recruitment to the sites of injury. ICOSL triggering by OPN induces cell migration, angiogenesis, and tumor metastatization (Raineri et al. 2020). Moreover, OPN has been identified as a cytokine which plays an important role in the development of type-1 immunity, through upregulation of macrophage IL-12 and downregulation of IL-10 expression, which promotes the Th1 and Th17 response (Ashkar et al. 2000; Iida et al. 2017; Lund et al. 2013). Via interactions with CD44 in Th cells, OPN enhances IL-17A and IFN-γ production and inhibits IL-4 and IL-10 which results in Th1 and Th17 polarization (Ashkar et al. 2000; Yan et al. 2018). It has been also documented that OPN acts as a pro-survival signal for DCs. In addition, via interactions with αv integrin and CD44, OPN regulates conventional DCs (cDCs) migration and functions. cDCs activation results in upregulation of IL-12, TNF-α and IFN-γ, and suppression of IL-27 production, which contributes to enhancement of the Th1 and Th17 immune response. Moreover, in plasmacytoid DCs, OPN enhances IFN-α synthesis (Del Prete et al. 2019). Likewise, it has been shown that OPN, with α4β1 and α9β1 integrins, contributes to neutrophil migration and activation (Koh et al. 2007). The available data indicate that OPN plays an important role in NK cell function—increases their activation, migration, expansion, as well as differentiation (Leavenworth et al. 2015).

## OPN in Kidney Transplantation

OPN has been found in renal tubular epithelium, ureteric buds and in interstitial cells in fetal kidney. In adult kidney OPN is expressed by the thick ascending limb of the loops of Henle and in collecting duct epithelium. Moreover, it has been revealed that OPN is secreted into urine (Xie et al. 2001). OPN’s role in the normal human kidney is not fully explained; however, it is believed that this protein is involved in tubulogenesis (Rogers et al. 1997). In addition, numerous studies have been conducted to evaluate OPN’s role in renal stone formation, but the obtained results are contradictory and inconclusive (Icer et al. 2018; Tsuji et al. 2007). It has been demonstrated that OPN expression is increased in numerous kidney diseases, including cancer, immunoglobulin A nephropathy, diabetic nephropathy, minimal change disease, focal and segmental glomerulosclerosis, membranous glomerulonephritis, and lupus nephritis (reviewed in Kaleta 2019a). In addition, some studies have suggested that OPN is associated with renal graft survival. Jin et al. (2013) measured serum OPN level in 77 patients before and after kidney transplantation (KTx) and in 78 healthy controls. The group demonstrated that in all KTx recipients pre-transplant serum OPN concentration was higher than in healthy controls. Moreover, elevated OPN level on day 0 and 7th after KTx was associated with the lower probability of rejection-free survival and was an independent predictor of acute rejection. In another study (Alchi et al. 2005) OPN renal expression and its correlation with clinical, laboratory, and histopathologic parameters in patients with or without acute renal allograft rejection was examined. In most biopsies of patients suffering acute rejection, OPN was highly expressed in the proximal tubular epithelium. In contrast, in biopsies of the non-rejecting KTx recipients, as well as in donor biopsies, OPN expression was weak or absent. Moreover, higher protein expression was associated with elevated interstitial monocyte infiltration and inflammation, which suggested a pathogenic OPN role in acute renal allograft rejection. In a similar study Rouschop et al. (2006) determined renal expression of OPN, its CD44 receptor, as well as the release of soluble OPN (sOPN) and sCD44 into the circulation during acute renal allograft rejection. It has been demonstrated that CD44 and OPN expression in biopsies with acute rejection was higher than in the non-rejecting group. Moreover, OPN expression positively correlated with the degree of interstitial inflammation. However, no association of CD44 and the Banff scores for tubulitis or interstitial inflammation has been found. It has been also revealed that plasma sCD44 concentration was higher in rejecting than in non-rejecting patients; however, the level of sOPN in plasma of patients from both groups was not significantly different. In a recent study by Mansour et al. (2021) the association between donor urine OPN and uromodulin (UMOD) concentration at the time of KTx and graft failure (GF), delayed graft function (DGF), and 6-month estimated glomerular filtration rate was analyzed. The group demonstrated that UMOD levels were lower with increasing severity of acute kidney injury. Moreover, higher urine OPN concentration in donors was associated with lower risk of DGF and GF, which suggests a protective role of this protein in graft function.

Most of the studies suggested that elevated OPN expression in kidneys or higher serum OPN level can be a predictor of acute rejection episodes. However, more research is required to understand the role of this protein in renal allograft dysfunction.

## OPN in Heart Transplantation

In physiological conditions OPN expression in cardiac myocytes, fibroblasts, and microvascular endothelial cells is low (Singh et al. 2014); however, it increases in several cardiovascular pathologies. Similarly, elevated OPN serum levels have been found in some cardiovascular diseases; therefore, this protein has been considered as a potential biomarker and mediator in atherosclerosis (Momiyama et al. 2010), myocardial infarction (Coculescu et al. 2019), as well as heart failure (Stawowy et al. 2002).

Nowadays, heart transplantation (HTx) is a gold standard treatment for patients with advanced heart failure (Kittleson and Kobashigawa 2017). Despite the progress in donor–recipient matching, and modern immunosuppression, graft rejection is still a leading cause of death after HTx. The most common cause of late graft failure is cardiac allograft vasculopathy (CAV) (Kennel and Schulze 2015). Endomyocardial biopsy (EMB) is the standard method for early detection of acute cellular rejection; however, EMB is more relevant to CAV prognosis than diagnosis, because it has low sensitivity due to the exclusion of microvasculature (Hiemann et al. 2007). Therefore, a lot of research is carried out to establish precise biomarkers in cardiac biopsy tissue as well as in blood which can be used for graft rejection screening (Kennel and Schulze 2015). Considering the role of OPN in various cardiovascular pathologies, as well as inflammatory conditions, it has been proposed that this protein may be implicated in HTx outcomes. So far only a few studies have been conducted in this field. Schipper et al. (2011) analyzed plasma OPN level in patients with end-stage heart failure before and after left ventricular assist device (LVAD) implantation and HTx. It has been demonstrated that OPN plasma concentration in patients with heart failure was approximately 5.5 times higher than in healthy controls, but it did not differ significantly before and after LVAD implantation. In addition, it has been found that OPN plasma concentration after HTx decreased significantly to levels observed in the control group. In another study Irion et al. (2020) analyzed the expression of nuclear OPN in cardiac tissue from 20 heart transplant patients receiving retransplantation. Moreover, the number of cells (including cardiomyocytes and non-cardiomyocytes) with nuclear OPN has been evaluated. The study demonstrated that 15/20 patients from the studied group had CAV, and more than 86% of them expressed nuclear OPN in myocardial tissue. In addition, 80% (4/5) of non-CAV patients, also expressed nuclear OPN. It has been found that nuclear OPN is expressed mainly in cardiomyocytes in both CAV and non-CAV patients (60% of cardiomyocytes vs. 40% of non-cardiomyocytes in CAV patients; 62% of cardiomyocytes vs. 38% of non-cardiomyocytes in non-CAV patients). The authors suggested that nuclear OPN presence in cardiac biopsies could act as an invasive marker for retransplantation. It is also possible that nuclear OPN expression may occur before or simultaneously with the development of CAV in transplant patients, which could be used for disease screening during endomyocardial biopsy in combination with coronary angiography.

## OPN in Liver Transplantation

In a normal liver, OPN is expressed at a low level in hepatocytes, stellate cells and hepatic macrophages (Nagoshi 2014). However, in pathological conditions, during inflammation, carcinogenesis, as well as fibrosis, OPN expression is upregulated (Bruha et al. 2020). Similarly, the serum OPN level has been found to be elevated and it can serve as a predictive marker for various liver diseases, including non-alcoholic steatohepatitis (NASH) (Syn et al. 2012), alcoholic liver disease (Patouraux et al. 2012), chronic hepatitis B and C (Huang et al. 2010; Zhao et al. 2008), and hepatocellular carcinoma (HCC) (Duarte-Salles et al. 2016; Gotoh et al. 2002; Shang et al. 2012).

One of the most common indications for liver transplantation (LTx) is cirrhosis and HCC caused by hepatitis C virus (HCV) and hepatitis B virus infection (El-Serag 2012; Ferrarese et al. 2016; Viveiros et al. 2017), and NASH (Syn et al. 2012). OPN has been recently considered as a prognostic factor for LTx. Cabiati et al. (2017) measured three OPN isoforms (named OPN-a, OPN-b, and OPN-c) mRNA expression and protein level in the plasma and liver tissue of patients with HCV-positive HCC undergoing LTx and in liver donors. The correlation of OPN-a, OPN-b, and OPN-c, and Notch-1, IV-Collagen-7s domain, IL-6 and TNF-α was also evaluated. It was demonstrated that OPN-a, OPN-b, and OPN-c mRNA expression was higher in HCC patients for LTx than in donors. Moreover, OPN-a, OPN-b, and OPN-c correlated with IV-Collagen-7s and Notch-1 (only isoform OPN-c). Similar associations have been found for OPN plasma and liver concentrations. OPN-a, OPN-b, and OPN-c concentrations in plasma and liver were higher in recipients with respect to donors; however, the results were not statistically significant. In addition, the group observed a reduction of OPN-a plasma concentration at 6 months after LTx, and, therefore, suggested an important role of this protein in patient screening after LTx. In another study immunohistochemical investigation of OPN expression was carried out in HCC patients undergoing LTx (Sieghart et al. 2011). Importantly, 65% of the participants were outside the Milan criteria. OPN expression was detected in 32% of the HCC patients. The overall post-transplant survival was significantly longer in patients without OPN expression. Likewise patients beyond the Milan criteria without OPN expression had better prognosis (tumor recurrence occurred in 43% of patients without and 70% of patients with OPN expression). The results strongly suggest that OPN reduces patient survival after LTx for HCC.

## OPN in Hematopoietic Stem Cell Transplantation

Allogenic HSCT is an important therapeutic option for numerous malignant and non-malignant diseases, including multiple myeloma, Hodgkin’s and non-Hodgkin’s lymphoma, leukemias, solid tumors, aplastic anemia, severe combined immune deficiency syndrome, and many others (Einhorn et al. 2007; Iftikhar et al. 2020; Pai et al. 2014; Peinemann et al. 2011; Rondelli et al. 2014; Yanada et al. 2006). The major complication of HSCT is acute or chronic GVHD (Ghimire et al. 2017). Acute GVHD occurs in 17–31% of patients after HSCT and develops within a few weeks after transplantation (Beckman et al. 2021). It usually targets the skin, liver, gastrointestinal tract, eyes, mouth, genitalia, and lungs (Harris et al. 2016). The disease develops when the recipient antigen-presenting cells are activated by the conditioning regimen and production of proinflammatory cytokines. Next, donor T cells are activated to recognize recipient antigens, migrate to target tissues and induce apoptosis (Ghimire et al. 2017). Although numerous studies confirmed the immunomodulatory properties of OPN and its probable role in transplantation, it is not fully elucidated whether this protein participates in the pathogenesis and course of GVHD after HSCT. To date only a few studies have been conducted in this field and gave opposite results. Zhao et al. (2011) analyzed the role of OPN in CD8^+^ T cell-mediated GVHD in an allogeneic HSCT mouse model of human GVHD. It has been shown that during GVHD, the OPN level in recipients was elevated and associated with increased migration and infiltration of CD8^+^ T cells. Anti-OPN antibody treatment reduced the number of infiltrated donor CD8^+^ T cells, their viability and activation, as well as the symptoms of GVHD. The above results suggest that OPN plays a significant role in GVHD pathogenesis. However, opposite results have been obtained in a study of Kawakami et al. (2017) who investigated the role of OPN in acute gastrointestinal GVHD in mice. It has been shown that in OPN knockout mice, infiltration of CD4^+^ and CD8^+^ T cells in the colon and small intestine was increased and the gastrointestinal GVHD score was elevated. Moreover, in the absence of OPN, both, the expression of proinflammatory cytokines—IL-17A, IL-18, IFN-γ, and TNF-α—as well as the number of apoptotic epithelial cells were elevated. Previously (Kaleta 2019b) analyzed the impact of OPN on the proliferation of human peripheral blood mononuclear cells (PBMCs) in a mixed lymphocyte reaction (MLR) has been analyzed. The MLR is an in vitro model of evaluation donor-specific alloreactivity. The MLR done before HSCT helps to evaluate the matching between donor and recipient and to assess the GVHD risk (Sayılan Şen et al. 2010). It has been shown that OPN dose-dependently increased the proliferation of alloactivated PBMCs, which suggests that this protein can become a predictive marker of GVHD in HSCT patients (Kaleta 2019b).

## OPN in Lung Transplantation

Lung transplantation (LuTx) is a complex procedure with high risk of perioperative morbidity and mortality; however, it has become a standard treatment option in patients with end-stage lung diseases (Yeung and Keshavjee 2014). Despite advances in both the selection of donor–recipient pairs, as well as in treatment of recipients, acute cellular rejection, antibody-mediated rejection and lymphocytic bronchiolitis are important risk factors for the development of chronic lung allograft dysfunction and patients deaths (Parulekar and Kao 2019). Therefore, the discovery of biomarkers that will help with the early identification of susceptible recipients, the assessment of the graft dysfunction risk, and the development of targeted therapies is one of the future lines of research. It has been demonstrated that OPN is one of the most highly expressed proteins in the lung of patients with numerous lung disorders (O’Regan 2003). Mura et al. (2019) found that OPN was one of the five most upregulated gene in lungs of patients with severe pulmonary arterial hypertension who underwent LuTx. Moreover, high OPN expression correlated with disease severity. Similar results have been obtained in a study of Gui et al. (2020) who measured OPN expression in lung transplant specimens of idiopathic pulmonary fibrosis patients. Immunohistochemical staining revealed high OPN expression in the alveolar epithelial cells lining the honeycomb space and alveolar macrophages accumulating in interalveolar spaces adjacent to the fibrotic lesion. The obtained results suggest that OPN can be a potential biomarker associated with poor graft function.

## Conclusion

To the best of our knowledge, this is the first study that comprehensively summarizes the role of OPN in kidney, heart, liver, hematopoietic stem cell, and lung transplantation. Most of the results, which are summarized in Table 1, have demonstrated an association of elevated OPN mRNA and protein expression and poorer graft function; however, a few studies gave opposite results suggesting its protective role. Therefore, future investigation is needed to verify if OPN can serve as a biomarker of organ and HSC transplantation. The knowledge about novel, non-invasive markers will promote our understanding of the mechanisms underlying graft dysfunction and post-transplant mortality. In addition, such knowledge may be helpful in the development of new treatment strategies and identification of recipients with increased risk of allograft failure.

**Table 1:** Main studies of association between osteopontin (OPN) and transplantation

Procedure	Reference	OPN pathogenic role	Comments
KTx	Jin et al. (2013)	Yes	In KTx recipients pre-transplant serum OPN level was higher than in healthy controls. Elevated OPN level on day 0 and 7th after KTx was associated with the lower probability of rejection-free survival and was an independent predictor of AR
	Alchi et al. (2005)	Yes	OPN expression in kidney biopsies of patients with AR was high and correlated with elevated interstitial monocytes infiltration, and inflammation
	Rouschop et al. (2006)	Yes/No	Renal OPN and CD44 expression was higher in KTx patients with AR than in non-rejecting group and correlated with the degree of interstitial inflammation. Soluble CD44 plasma concentration was higher in rejecting than in non-rejecting patients; however, soluble OPN plasma level was similar in both groups
	Mansour et al. (2021)	No	Elevated urine OPN level and OPN expression in kidney donors was associated with lower risk of delayed graft function and graft failure
HTx	Schipper et al. (2011)	Yes	OPN plasma level was higher in patients with heart failure than in healthy controls and decreased after HTx
	Irion et al. (2020)	Yes	OPN expression in cardiac tissue of patients with CAV who received retransplantation was high
LTx	Cabiati et al. (2017)	Yes	OPN plasma level and OPN expression in liver tissue of patients with HCV-positive HCC undergoing LTx was higher than in healthy controls and correlated with thrombin, 7 s-collagen and Notch-1. A significant reduction of OPN plasma levels was observed at 6 months after LTx
	Sieghart et al. (2011)	Yes	OPN expression in HCC patients undergoing LTx was high. The overall post-transplant survival was significantly longer in patients without OPN expression. Likewise patients beyond the Milan criteria without OPN expression had better prognosis
HSCT	Zhao et al. (2011)	Yes	During CD8^+^ T cell-mediated GVHD in allogeneic HSCT mouse model, OPN level in recipients was elevated and associated with increased migration and infiltration of CD8^+^ T-cells. The anti-OPN Ab treatment reduced the number of infiltrated donor CD8^+^ T-cells, their viability and activation, as well as the symptoms of GVHD
	Kawakami et al. (2017)	No	In OPN knockout mice, the infiltration of CD4^+^ and CD8^+^ T cells in the colon and small intestine of was increased and the gastrointestinal GVHD score was elevated. In the absence of OPN, the expression of proinflammatory cytokines: IL-17A, IL-18, IFN-γ, TNF-α, as well as the number of apoptic epithelial cells was elevated
	Kaleta (2019a, b)	Yes	OPN dose-dependently increased the proliferation of alloactivated human PBMCs in a MLR
LuTx	Mura et al. (2019)	Yes	OPN was one of the five most upregulated gene in lungs of patients with severe PAH who underwent LuTx. High OPN expression correlated with disease severity
	Gui et al. (2020)	Yes	OPN expression in a lung transplant specimens of idiopathic pulmonary fibrosis patients was high

*Ab* antibody; *AR* acute rejection; *CAV* cardiac allograft vasculopathy; *GVHD* graft versus host disease; *HCC* hepatocellular carcinoma; *HCV* hepatitis C virus; *HSCT* hematopoietic stem cell transplantation; *HTx* heart transplantation; *IFN* interferon; *IL* interleukin; *KTx* kidney transplantation; *LTx* liver transplantation; *LuTx* lung transplantation; *MLR* mixed lymphocyte reaction; *OPN* osteopontin; *PAH* pulmonary arterial hypertension; *PBMCs* peripheral blood mononuclear cells; *TNF* tumor necrosis factor
